# Supplementary material for: Neutrophil to Lymphocyte Ratio as a Biomarker for the Prediction of Cancer Outcomes and Immune-Related Adverse Events in a CTLA-4-Treated Population
Source: Cancers (Basel). 2025 Jun 17;17(12):2011. doi: 10.3390/cancers17122011 (PMC12190284; doi:10.3390/cancers17122011)
Supplement: Supplementary file 1 [file cancers-17-02011-s001.zip › Supplemental Table S1.pptx]

## Slide 1
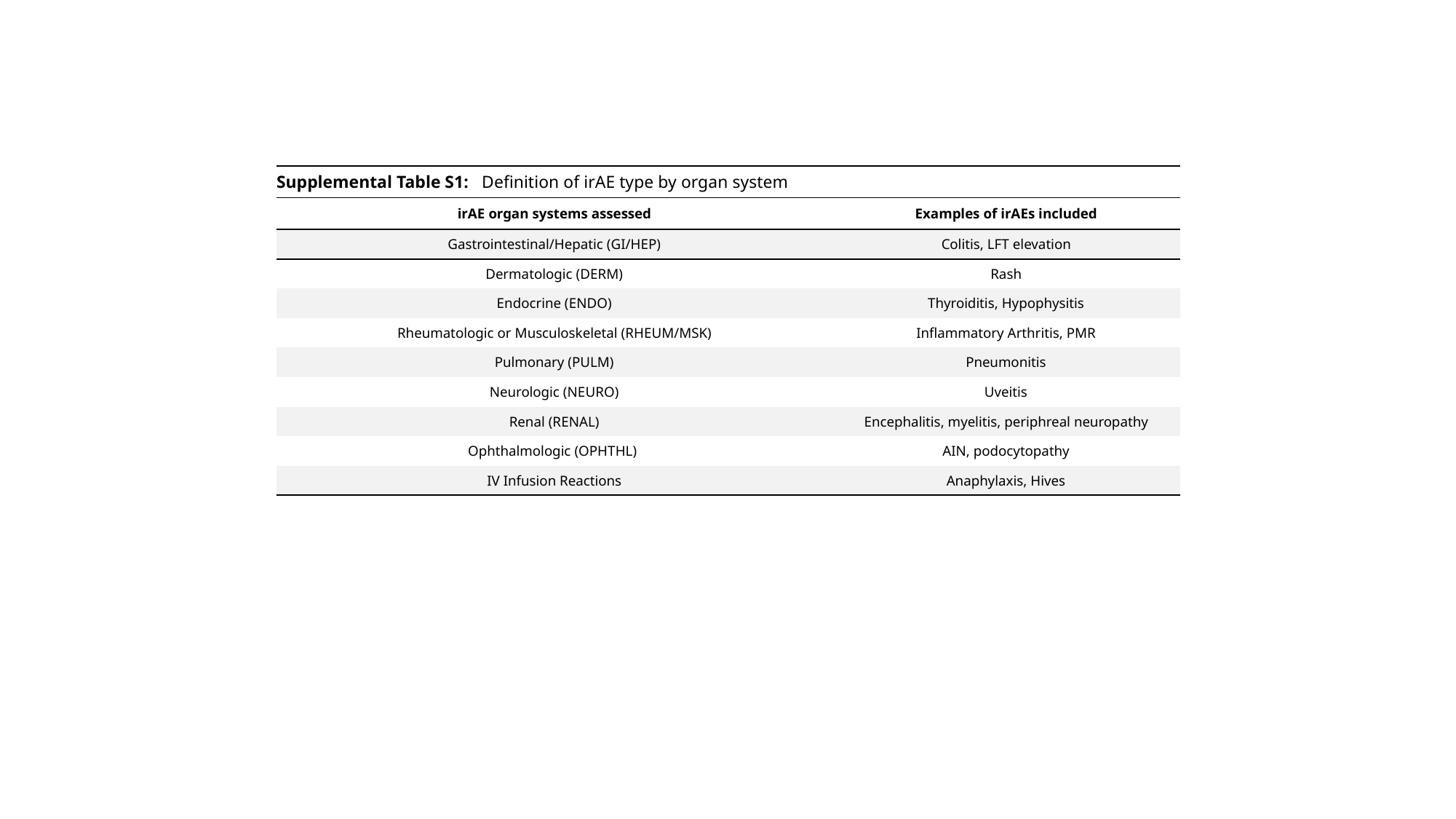

| Supplemental Table S1:   Definition of irAE type by organ system | |
| --- | --- |
| irAE organ systems assessed | Examples of irAEs included |
| Gastrointestinal/Hepatic (GI/HEP) | Colitis, LFT elevation |
| Dermatologic (DERM) | Rash |
| Endocrine (ENDO) | Thyroiditis, Hypophysitis |
| Rheumatologic or Musculoskeletal (RHEUM/MSK) | Inflammatory Arthritis, PMR |
| Pulmonary (PULM) | Pneumonitis |
| Neurologic (NEURO) | Uveitis |
| Renal (RENAL) | Encephalitis, myelitis, periphreal neuropathy |
| Ophthalmologic (OPHTHL) | AIN, podocytopathy |
| IV Infusion Reactions | Anaphylaxis, Hives |
