# Supplementary material for: Neutrophil to Lymphocyte Ratio as a Biomarker for the Prediction of Cancer Outcomes and Immune-Related Adverse Events in a CTLA-4-Treated Population
Source: Cancers (Basel). 2025 Jun 17;17(12):2011. doi: 10.3390/cancers17122011 (PMC12190284; doi:10.3390/cancers17122011)
Supplement: Supplementary file 1 [file cancers-17-02011-s001.zip › Supplemental Table S2.pptx]

## Slide 1
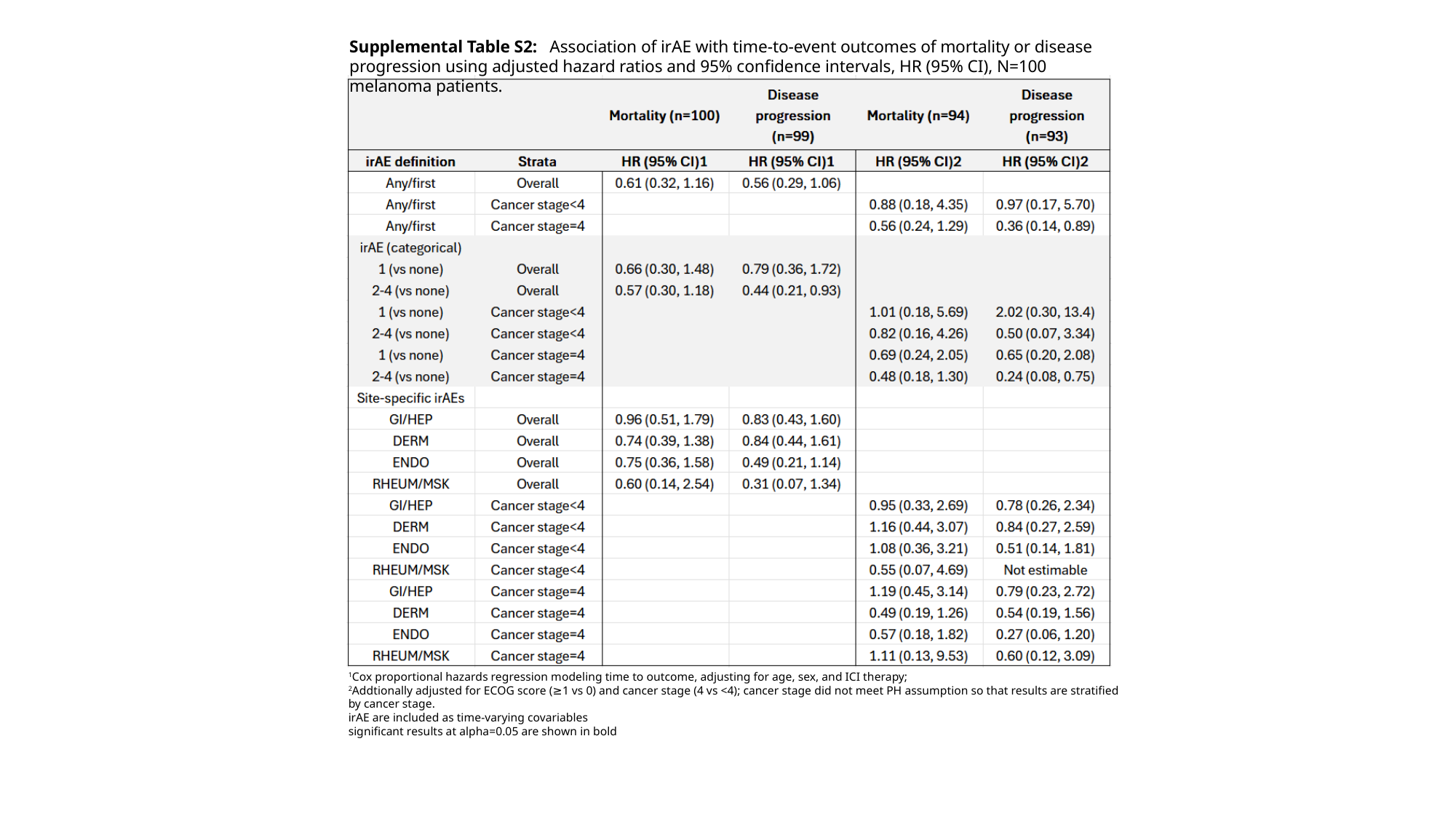

Supplemental Table S2: Association of irAE with time-to-event outcomes of mortality or disease progression using adjusted hazard ratios and 95% confidence intervals, HR (95% CI), N=100 melanoma patients.
1Cox proportional hazards regression modeling time to outcome, adjusting for age, sex, and ICI therapy;
2Addtionally adjusted for ECOG score (≥1 vs 0) and cancer stage (4 vs <4); cancer stage did not meet PH assumption so that results are stratified by cancer stage.
irAE are included as time-varying covariables
significant results at alpha=0.05 are shown in bold
