# Supplementary material for: Neutrophil to Lymphocyte Ratio as a Biomarker for the Prediction of Cancer Outcomes and Immune-Related Adverse Events in a CTLA-4-Treated Population
Source: Cancers (Basel). 2025 Jun 17;17(12):2011. doi: 10.3390/cancers17122011 (PMC12190284; doi:10.3390/cancers17122011)
Supplement: Supplementary file 1 [file cancers-17-02011-s001.zip › Supplemental Table S3.pptx]

## Slide 1
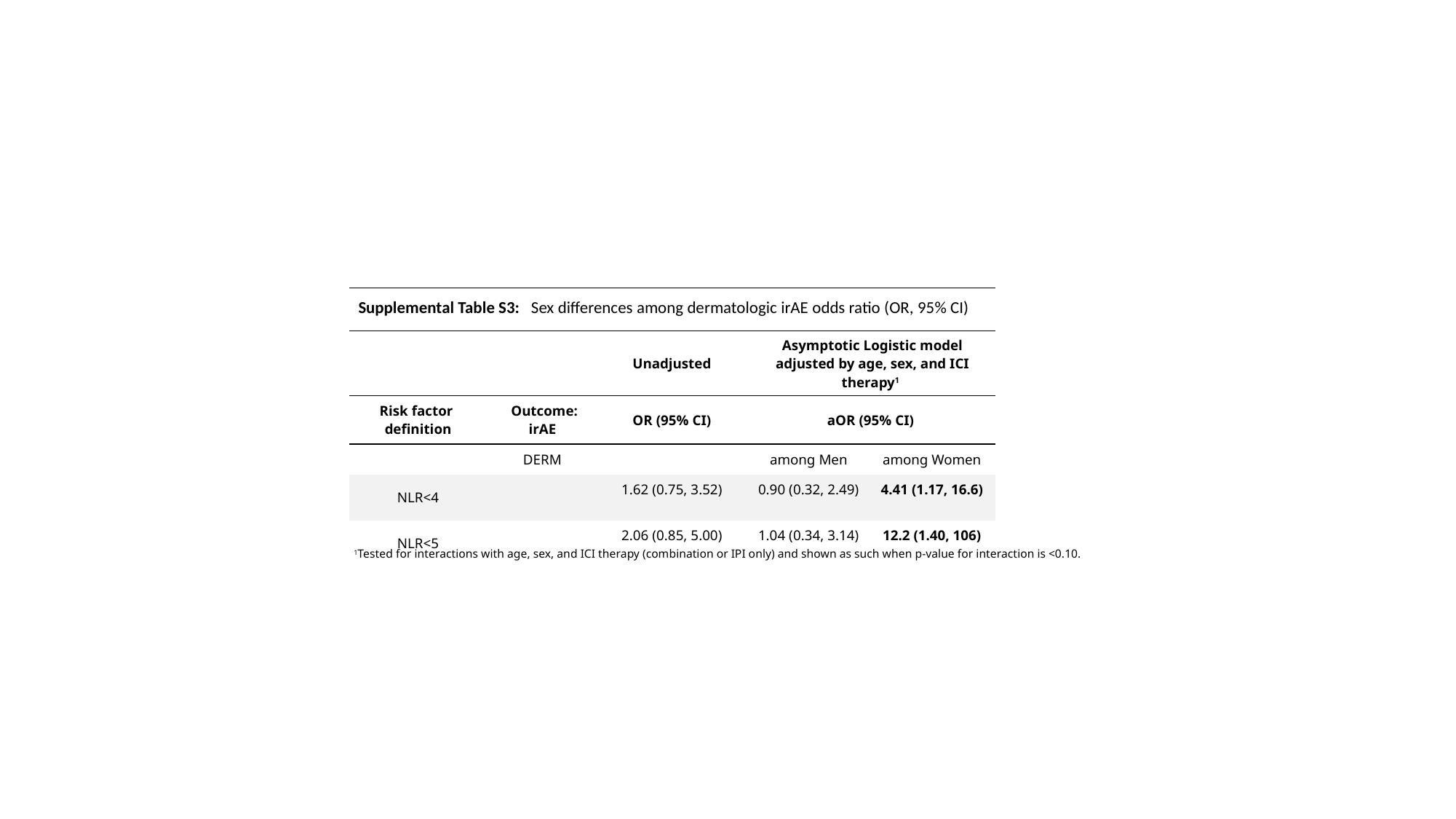

| Supplemental Table S3:   Sex differences among dermatologic irAE odds ratio (OR, 95% CI) | | | | |
| --- | --- | --- | --- | --- |
| | | Unadjusted | Asymptotic Logistic model adjusted by age, sex, and ICI therapy1 | |
| Risk factor   definition | Outcome: irAE | OR (95% CI) | aOR (95% CI) | |
| | DERM | | among Men | among Women |
| NLR<4 | | 1.62 (0.75, 3.52) | 0.90 (0.32, 2.49) | 4.41 (1.17, 16.6) |
| NLR<5 | | 2.06 (0.85, 5.00) | 1.04 (0.34, 3.14) | 12.2 (1.40, 106) |
1Tested for interactions with age, sex, and ICI therapy (combination or IPI only) and shown as such when p-value for interaction is <0.10.
