# Supplementary material for: Neutrophil to Lymphocyte Ratio as a Biomarker for the Prediction of Cancer Outcomes and Immune-Related Adverse Events in a CTLA-4-Treated Population
Source: Cancers (Basel). 2025 Jun 17;17(12):2011. doi: 10.3390/cancers17122011 (PMC12190284; doi:10.3390/cancers17122011)
Supplement: Supplementary file 1 [file cancers-17-02011-s001.zip › Supplemental Table S4.pptx]

## Slide 1
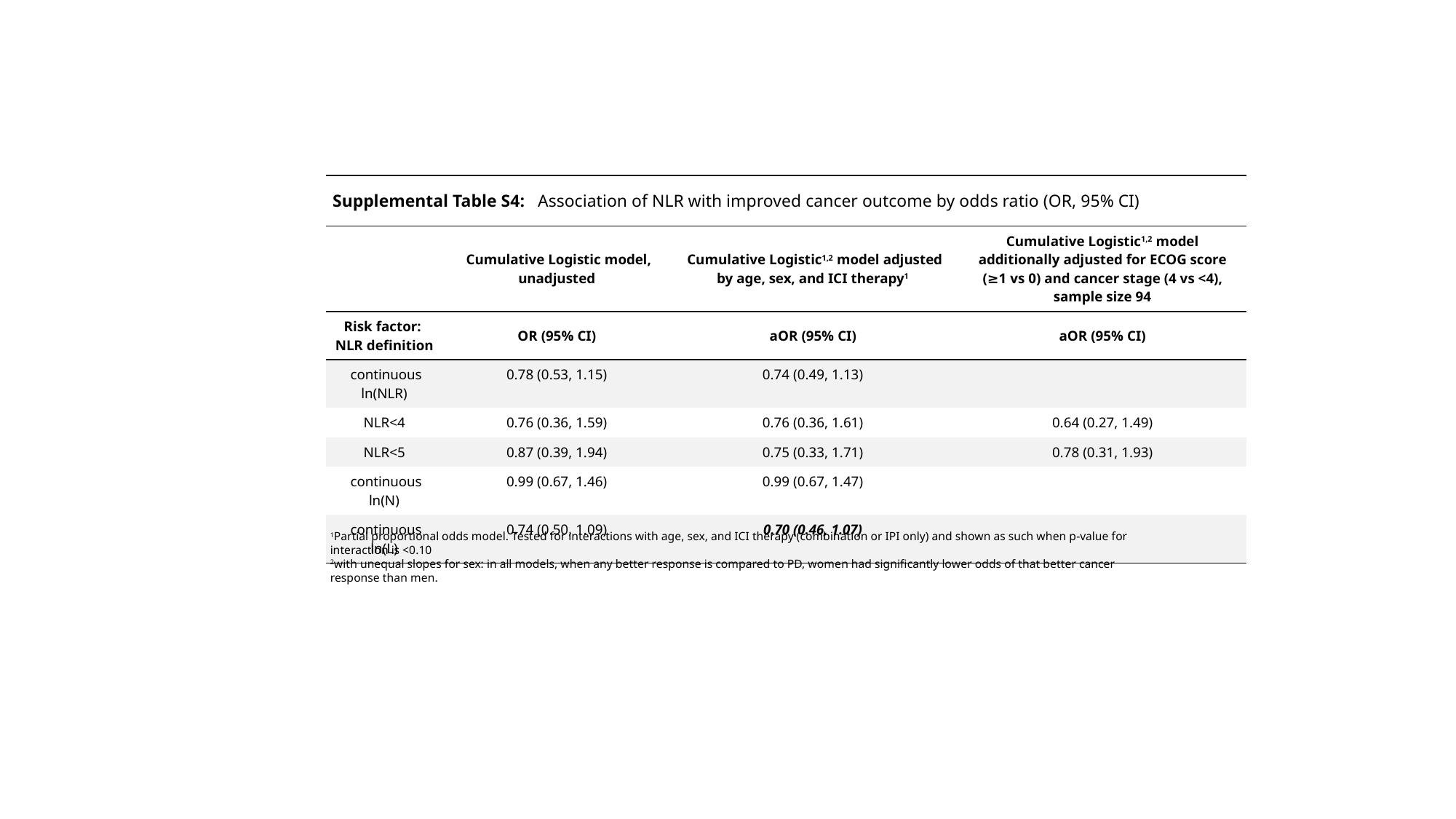

| Supplemental Table S4:   Association of NLR with improved cancer outcome by odds ratio (OR, 95% CI) | | | |
| --- | --- | --- | --- |
| | Cumulative Logistic model, unadjusted | Cumulative Logistic1,2 model adjusted by age, sex, and ICI therapy1 | Cumulative Logistic1,2 model additionally adjusted for ECOG score (≥1 vs 0) and cancer stage (4 vs <4), sample size 94 |
| Risk factor:   NLR definition | OR (95% CI) | aOR (95% CI) | aOR (95% CI) |
| continuous ln(NLR) | 0.78 (0.53, 1.15) | 0.74 (0.49, 1.13) | |
| NLR<4 | 0.76 (0.36, 1.59) | 0.76 (0.36, 1.61) | 0.64 (0.27, 1.49) |
| NLR<5 | 0.87 (0.39, 1.94) | 0.75 (0.33, 1.71) | 0.78 (0.31, 1.93) |
| continuous ln(N) | 0.99 (0.67, 1.46) | 0.99 (0.67, 1.47) | |
| continuous ln(L) | 0.74 (0.50, 1.09) | 0.70 (0.46, 1.07) | |
1Partial proportional odds model. Tested for interactions with age, sex, and ICI therapy (combination or IPI only) and shown as such when p-value for interaction is <0.10
2with unequal slopes for sex: in all models, when any better response is compared to PD, women had significantly lower odds of that better cancer response than men.
